# Saturated Emission States in Fluorescent Nanostructured Media: The Role of Competition between the Stimulated Emission and Radiation Losses in the Local Emitters of Fluorescence

**DOI:** 10.3390/nano12142450

**Published:** 2022-07-17

**Authors:** Dmitry Zimnyakov, Sergey Volchkov, Leonid Kochkurov, Alexander Dorogov

**Affiliations:** 1 Physics Department, Yury Gagarin State Technical University of Saratov, 410054 Saratov, Russia; volchkov93@bk.ru (S.V.); lkochkurov@gmail.com (L.K.); sanek_9993@mail.ru (A.D.); 2 Precision Mechanics and Control Institute, Russian Academy of Sciences, 24 Rabochaya Str., 410024 Saratov, Russia

**Keywords:** fluorescence, nanostructured dispersive media, random lasing, spectral quality, saturation

## Abstract

A fundamental limitation of the spectral response of laser-pumped fluorescent nanostructured media was considered in terms of a probabilistic model establishing the relationship between the enhancement factor of a spectral quality and characteristic propagation and amplification scales of pump light and fluorescence in the medium. It was shown that the minimum spectral width of fluorescent response of the pumped medium is limited by competition between the stimulated emission and radiation losses in microscopic fluorescence emitters associated with the speckles randomly modulating the pumping light field. Theoretical results were compared to the experimental data on the spectral properties of the fluorescent response of laser-pumped nanostructured “anatase nanoparticles—dye solutions” systems with various structural and optical properties. Rhodamine 6G and 4-(dicyanomethylene)-2-methyl-6-(p-dimethylaminostyryl)-4H-pyran (DCM) were applied as fluorescent components in the examined systems with various scatter volume fractions, which were pumped by pulse-periodic laser radiation with various intensities at 532 nm. Comparison showed a fair agreement between the theoretical and experimental results.

## 1. Introduction

Features of fluorescence excitation in laser-pumped random media have been the subject of numerous experimental and theoretical studies over the past 30 years. This research activity was particularly inspired by expectation of an application of a new type of cavityless laser devices known as random lasers. The idea of random lasing in structurally disordered active media began its existence with a pioneering work of V. S. Letokhov [1] and gave rise to abundance of publications devoted to various aspects of this phenomenon (see., e.g., [2,3,4,5,6,7,8,9,10,11,12,13,14,15,16,17,18,19,20]). However, all the examined random lasing systems based on disordered nano- or micro-structured media performed several orders of magnitude worse than conventional cavity-containing laser systems in terms of the spectral properties of the emission such as the spectral quality $\Psi_{sp}=\bar{\lambda }/\Delta \lambda$ or the β-factor. Here, $\bar{\lambda }$ and Δλ are an average wavelength and FWHM value of the emission spectrum, and the definition of the β-factor as applied in random lasing systems is given in [9]. In addition to the low temporal coherence, the spatial coherence of radiation outgoing from such systems also turns out to be very low. These key factors significantly limit the widespread use of random lasers in various modern technologies. Nevertheless, currently diagnostic applications of the random lasing phenomenon in material science and biomedicine [21,22,23,24] are among modern research trends in photonics.

It should be noted that the use of dispersive nanostructured nanomaterials as strongly scattering matrix media in random lasing systems may be preferable compared to coarser-grained media. This is primarily due to the low values of the scattering anisotropy g and the mean transport free path $l^{*}$ [25] of the pump light and fluorescence propagation in such systems. In turn, this provides favorable conditions for achieving high pump energy densities in the volume of the pumped medium.

A general feature in the behavior of such systems at sufficiently high levels of external pumping is abrupt narrowing of the fluorescence spectrum with a relatively small increase in the pump intensity (a typical threshold-like behavior). This threshold-like character of the dependence of the fluorescence spectral quality $\Psi_{sp}$ on the pump intensity is interpreted as a manifestation of the transition from the spontaneous emission mode in the pumped system to the random lasing mode. In addition, another general property of the pump-stimulated fluorescence response of the fluorescent random media manifested in numerous experiments on random lasing (see, e.g., [2,5,15,16]) is the practically unchangeable spectral properties of outgoing radiation over a wide range of pump intensities significantly exceeding the conditional random lasing threshold $I_{p,th}$. This feature allows us to consider it as a direct manifestation of a pumped system transitioning into a certain saturated quasi-equilibrium state. Note that these saturated states of fluorescent random media pumped at high intensities relate only to the spectral properties (spectral half-widths and spectral qualities of emission) but not to energetic characteristics of the fluorescence output. In particular, a wavelength-integrated fluorescence energy flux from the pumped medium typically exhibits a close-to-proportional increase with an increasing pump intensity above the threshold.

From general considerations, it can be assumed that the transition of pumped systems to their saturated states is associated with competition of various processes in the system, leading to both an increase and a decrease in the emission rate. Accordingly, the saturated quasi-equilibrium states are achieved under the condition of mutual compensation of these processes. Note that the effect of saturation of the fluorescence spectral quality in the case of random lasing at high pump intensities has not yet been discussed in detail.

Previously [26], a kinetic model was considered for the conversion of the pump radiation into fluorescence in random media, which takes into account the granular structure of the pump light field. This granular structure is the result of stochastic interference of partial contributions to the pump light field, which propagate into the pumped medium along uncorrelated random traces and form a random ensemble of bulk speckles. Each speckle acts as a local microscopic emitter of the pump-induced fluorescence, and the fluorescence output of the pumped medium is formed due to incoherent summation of the contributions from various statistically independent speckle-associated local emitters in the pumped volume. Note that this approach to describing the conversion of pump radiation into fluorescence in random media is fundamentally different from another widely used approach, which is based on the application of the diffusion approximation of the radiative transfer theory (see, e.g., [6,9,27]). Accordingly, the latter concept does not take into account the granularity of the pump field, which arises as a result of significant excess in the coherence length of the pump radiation over a characteristic scale of its propagation in the medium. In nanostructured random media, the characteristic size of these speckle-associated emitters is expected at a sub-micrometer or even nanometer scale because of the close-to-isotropic scattering mode of pump radiation in these media. An ensemble-averaged cross section of radiation losses in the local speckle-associated fluorescence emitters, which was introduced in the framework of the kinetic model [26], is one of the key parameters determining the dynamics of the fluorescence response during the pumping procedure. In turn, this introduced parameter should determine, along with other emission parameters, the condition for the transitions to saturation in pumped nanostructured media.

The goal of this work is to consider experimentally observed saturation of fluorescence spectral characteristics and interpret their extreme values in the case of laser-pumped nanostructured media at high pump intensities. The consideration is carried out in terms of the fundamental relationship between the key parameters of fluorescence emission at the microscopic level (various cross sections characterizing the emission process, including the cross section of radiative losses in speckle-associated local emitters) and the characteristic scales of fluorescence propagation and amplification in the medium. Theoretical results of the analysis of extreme states of fluorescence emission, obtained using the developed probabilistic model of stationary fluorescence transfer in pumped media, are compared to the experimental data for laser-pumped nanostructured model media with various emission and structural properties.

## 2. Materials and Methods

### 2.1. Preparation of the Model Samples of Fluorescent Nanostructured Systems

The saturation of the spectral characteristics of fluorescence emission from nanostructured random media with an increasing laser pump intensity was experimentally studied using specially prepared model samples. These samples consisted of scattering randomly inhomogeneous matrices doped with laser dye solutions. Anatase nanoparticles (product #637254 of Sigma-Aldrich Co. (St. Louis, MO, USA)), polydisperse nanopowders with the average size of the particles being less than 25 nm) were used to prepare two types of scattering systems with significantly different volume fractions of scattering centers and, accordingly, values of the optical transport parameters. The samples of the first type were layers of densely packed nanoparticles, which were doped by dye solutions. The second group of samples included suspensions of nanoparticles in dye solutions; in the course of the experiments, samples with various volume fractions ρ of particles in suspension varying in the range from 0 to ≈0.049 were examined.

Rhodamine 6G (R6G) and 4-(dicyanomethylene)-2-methyl-6-(p-dimethylaminostyryl)-4H-pyran (DCM) solutions in ethanol were applied as fluorescent components for the prepared model samples. The choice of these laser dyes was due to substantial differences in their absorption at the pump wavelength of 532 nm and their emission properties. In particular, the pump wavelength approximately corresponds to the maximum absorption of R6G and is far enough from the maximum absorption of DCM (see, e.g., [28,29]). Among other things, this leads to a more than tenfold difference in absorption cross sections of fluorophore molecules for R6G- and DCM-based model samples. In addition, the spontaneous fluorescence lifetimes of R6G and DCM molecules differ by approximately a factor of 4 ($\tau_{R6G}\approx$ 3.8 ÷ 4.0 ns [30,31,32] against $\tau_{DCM}\approx$ 1.0 ns [33]). The absorption coefficients $\mu_{a}$ of the solutions with various dye concentrations at 532 nm were preliminarily estimated using the measurements of their collimated transmittance in 1 mm thick flat cuvettes. On this basis, the mole fractions $c_{M}$ of the dyes in the solutions were selected in such a way as to provide sufficiently close values of absorption coefficients of the solutions used to prepare the R6G- and DCM-doped samples. Accordingly, the molar concentrations were chosen to be $c_{M,R6G}\approx$ 3.4 × 10^−3^ M and $c_{M,DCM}\approx$ 6.1 × 10^−2^ M, and the corresponding absorption coefficients of the dye solutions were approximately equal to $\mu_{a,R6G}\approx$ (190 ± 10) cm^−1^ and $\mu_{a,DCM}\approx$ (220 ± 12) cm^−1^. Further details of sample preparation and characterization [34,35,36,37,38,39,40,41,42,43] are described in the Supplementary Materials of this article.

The estimated values of the mean transport free path $l^{*}$ and effective refractive index $n_{ef}$ for the prepared samples, which were used for further theoretical modeling, are presented in Table 1. The effective refractive indices for the samples #2 were taken equal to those of the solvent (ethanol) because of the small ρ values of anatase nanoparticles.

### 2.2. Experimental Technique

**Figure 1:**
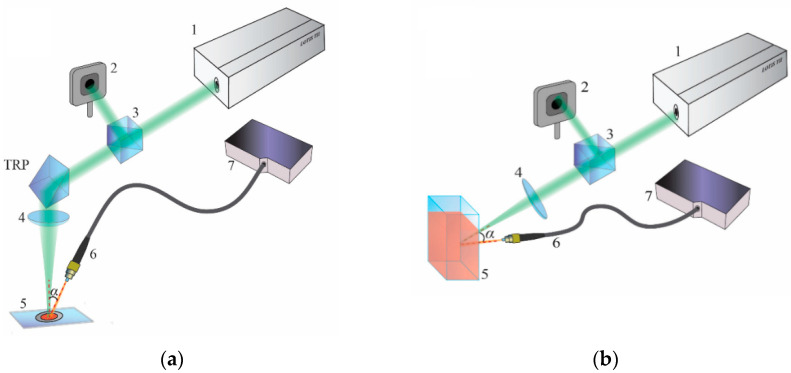
The scheme of the experimental setup. (**a**) Examination of samples #1 (layers); (**b**) examination of samples #2 (suspensions). 1—laser; 2—energy meter; 3—beam-splitter; 4—convex lens; 5—sample, 6—fiber-optic patch cord, 7—spectrometer; TRP—totally reflecting prism.

Experimental studies on the saturation effects of fluorescence spectral quality in model samples at high pump intensities were carried out using a typical scheme for excitation of random lasing (Figure 1). The second harmonic (532 nm) of the Q-switched YAG:Nd laser (the LS-2134 model from Lotis TII company (Minsk, Republic of Belarus), the pulse duration $\tau_{p}$ is 10 ns, the pulse repetition rate is 10 Hz, and the pulse energy $E_{p}$ varied from 0.5 mJ to 30 mJ) was used as pumping radiation. The pumping beam was focused using a convex lens with a focal length of 150 mm. Depending on their type, the samples under study were placed at different distances from the waist plane of the pumping beam in order to obtain the required range of pump intensities $I_{p}$. During the experiments, the samples were fixed on the XYZ translation stage (not shown in Figure 1), which provided a possibility for their positioning and displacement in the axial and lateral directions with a step of 10 µm. The pump intensity was evaluated at a given sample position as $I_{p}=4E_{p}/\pi d_{p}^{2}\tau_{p}$; the values of the light spot diameter $d_{p}$ depending on displacement of the irradiation plane with respect to the beam waist plane were preliminarily estimated using axial–lateral scanning of the waist zone by the Foucault knife with simultaneous measurements of transmitted pulse energy. In particular, the spot diameter $d_{p}\approx$ (1000 ± 50) µm was applied in the experiments with R6G-doped layers (the samples of the first type); in this case the samples were placed in the converging pump beam at a distance ≈29.5 mm above the defined position of the waist plane. Under changes in the pulse energy from 0.2 mJ to 7.0 mJ, this provided a range of available pump intensities from 2.5 × 10^6^ W/cm^2^ to 8.8 × 10^7^ W/cm^2^ which were sufficient for transition from a spontaneous fluorescence emission to saturation of the spectral quality in the random lasing mode for the examined R6G-doped layers. At the same time, the DCM-doped layers require significantly higher pump intensities to achieve a saturated state of spectral quality; therefore, they were located at a smaller distance (≈3.0 mm) from the waist plane. In this case, the laser spot size was equal to ≈(100 ± 10) µm. The samples of the second group (suspensions) were irradiated through a cell wall in such a way that the laser spot size at the glass–suspension interface was approximately equal to 100 µm. Suspensions were pumped with a fixed pump intensity of 8.8 × 10^7^ W/cm^2^. This value significantly exceeds the random lasing threshold for the R6G-doped samples #1 and is approximately 2.3 times larger than the threshold value for the DCM-doped samples #1. The choice of such a pumping mode is associated with the subsequent analysis of the effect of complete or partial saturation of the excited state population on the characteristic scale of fluorescence amplification in the pumped suspensions.

The energy of the laser pulses was measured by an energy–power meter (Maestro, Gentec Electro-Optics, Quebec, QC, Canada) using laser beam splitting by a 90:10 beam splitter; the instability of the energy values did not exceed 5%. During pumping, the fluorescent response of the studied samples was recorded using a fiber-optic patch cord (Ocean Optics P200-2-UV-VIS, Dunedin, FL, USA) connected to a spectrometer (Ocean Optics QE65000, Dunedin, FL, USA). The entrance end of the patch cord was located at a distance of 50 mm from the irradiated zone at an angle of ≈30° to the laser beam axis; preliminary adjustment of the angular position of the entrance end relative to the beam axis and the irradiated zone was performed to achieve the maximum fluorescent response acquired by the spectrometer.

To exclude photodegradation (the bleaching of a dye and the resulting decrease in the fluorescence output for the samples #1, radiation damage of the cuvette walls in the case of samples #2) of the examined samples in the irradiated zone as a result of a long-term laser irradiation, the fluorescence spectra were recorded at a given value of the pump pulse energy in accordance with the following procedure:

- At a fixed position, the samples were irradiated by sequences of five pulses with simultaneous acquisition of the fluorescence response by the spectrometer with an integration time equal to the duration of the sequences (500 ms);
- After that, the samples were translated in the lateral direction relative to the laser beam to a distance exceeding $d_{p}$, and the irradiation cycle was repeated;
- Finally, the fluorescence spectrum of the sample at a given pump pulse energy was obtained by the averaging of separate spectra captured at five different lateral positions of the sample.

## 3. Experimental Results

**Figure 2:**
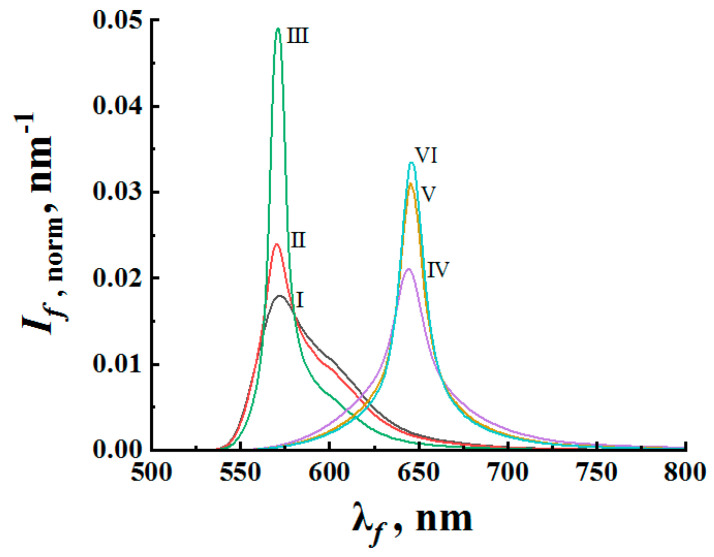
Evolution of the normalized fluorescence spectra with an increase in the pump intensity. I–III—R6G-doped samples #1; VI–VI—DCM-doped samples #1. $I_{p}\approx$1.0 × 10^6^ W/cm^2^ (I); 1.3 × 10^7^ W/cm^2^ (II); 3.9 × 10^7^ W/cm^2^ (III); 2.2 × 10^7^ W/cm^2^ (IV); 1.0 × 10^8^ W/cm^2^ (V); 1.4 × 10^8^ W/cm^2^ (VI).

Figure 2 illustrates the changes in the shape of the fluorescence spectra with increasing pump intensity $I_{p}$ for R6G- and DCM-doped samples of the first group (layers of densely packed nanoparticles). For evidence of the fluorescence spectra narrowing with increasing pump intensity, Figure 2 displays normalized values of the fluorescence intensity $I_{f,norm}\left(\lambda \right)=I_{f}\left(\lambda \right)/\int_{\lambda_{\min}}^{\lambda_{\max}}I_{f}\left(\lambda \right)d\lambda$ depending on the wavelength (here, $\lambda_{\min},\lambda_{\max}$ define the fluorescence band positions in the wavelength domain). Note that the random lasing threshold, traditionally determined by a twofold decrease in the half-width of the fluorescence spectrum, is significantly higher for DCM-saturated samples compared to R6G-saturated samples ($I_{p,th}^{DCM}\approx$ 3.6 × 10^7^ W/cm^2^ against $I_{p,th}^{R6G}\approx$ 9.0 × 10^6^ W/cm^2^). This is primarily due to a significantly smaller absorption cross section of pump radiation and, simultaneously, a significantly higher rate of transition from the excited state to the ground state for DCM molecules. Accordingly, this should lead to substantially lower values of the relative population of the excited state under the action of pump pulses in the case of DCM-doped layers. At the same time, the fluorescence spectra for both systems show a half-width saturation trend with an increasing pump intensity to values significantly exceeding $I_{p,th}$. This trend can be considered in terms of an enhancement factor of the spectral quality introduced as

(1) $$Q_{sp}\left(I_{p}\right)=\frac{\Delta \lambda \left(I_{p}\right)}{\Delta \lambda \left(0\right)}.$$

**Figure 3:**
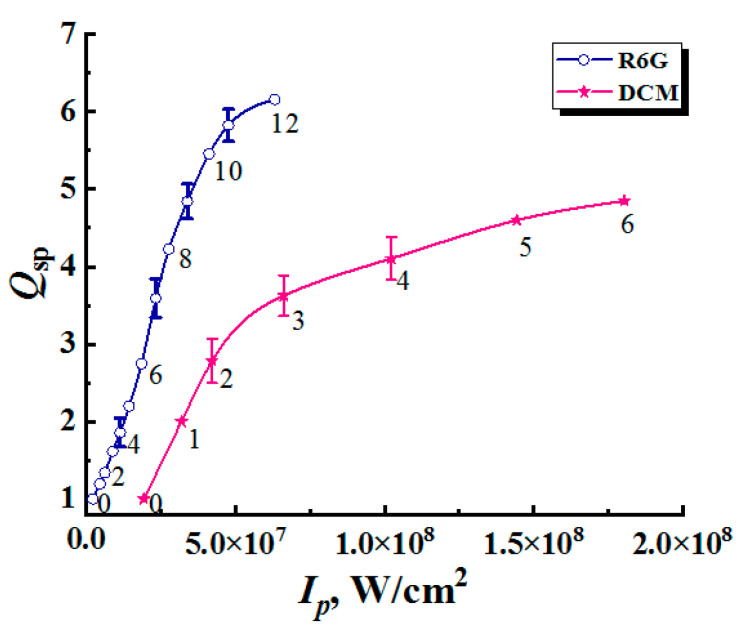
$Q_{sp}$ values against pump intensity for the R6G- and DCM-doped samples #1. Selectively shown error bars correspond to a confidence level of 0.9.

Here, $\Delta \lambda \left(I_{p}\right)$ is the FWHM value of the emission spectrum at the given pump intensity, and Δλ(0) corresponds to the case of a spontaneous emission mode at low pump intensities. Figure 3 displays the values of the introduced enhancement factor (1) against the pump intensity for samples #1.

**Figure 4:**
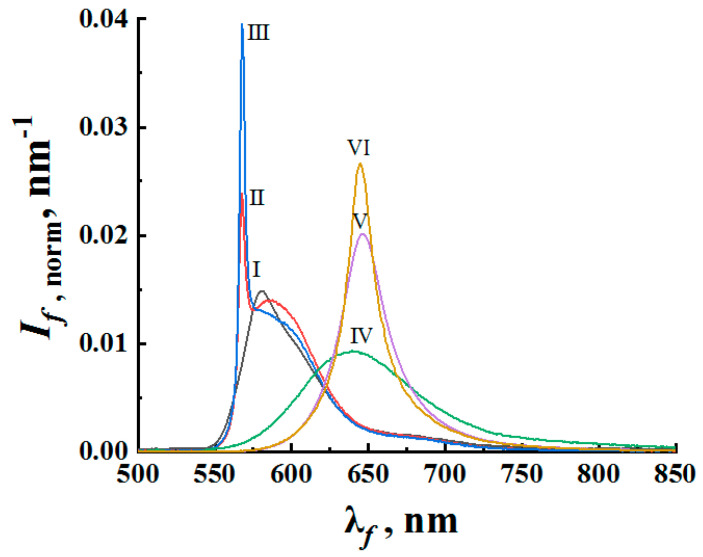
Evolution of the normalized fluorescence spectra with an increase in the scatter volume fraction at a fixed pump intensity. I–III—R6G-based samples #2; IV–VI—DCM-based samples #2 ($I_{p}=$ 8.8 × 10^7^ W/cm^2^). ρ= 0 (I, no particles); ρ≈ 0.0073 (II); ρ≈ 0.012 (III); ρ= 0 (IV, no particles); ρ≈ 0.014 (V); ρ≈ 0.026 (VI).

**Figure 5:**
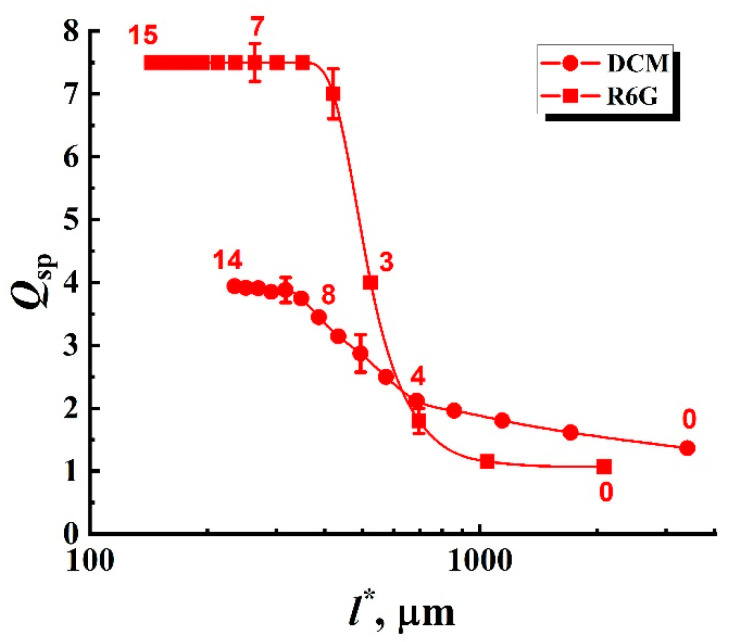
$Q_{sp}$ values against the MTFP values for the R6G- and DCM-based suspensions (samples #2). Selectively shown error bars correspond to a confidence level of 0.9.

Similarly, the obtained fluorescence spectra for samples #2 exhibit narrowing with a half-width saturation trend with an increase in the volume fraction of anatase nanoparticles in the suspensions (and, accordingly, a decrease in the mean transport free path of pump and fluorescence propagation in irradiated samples). Similar to Figure 2, Figure 4 displays characteristic changes in the shape of the fluorescence spectra of the samples #2 with an increase in the volume fraction of the nanophase (anatase particles) at a fixed value of $I_{p}\approx$ 8.8 × 10^7^ W/cm^2^. In addition to transverse displacement of the cuvette with the sample, mixing the suspension in the cuvette was carried out during the experiment after each cycle of five pump pulses. Figure 5 shows the values of the enhancement factor $Q_{sp}$ depending on the MTFP values for the average fluorescence wavelengths ${\bar{\lambda }}_{1}$ and ${\bar{\lambda }}_{2}$. The trend towards saturation of $Q_{sp}$ with decreasing $l^{*}$ is obvious (2).

The remarkable fact is that the R6G-based suspensions exhibit a blue shift (up to 13 nm, Figure 4) of the fluorescence spectral maximum in the random lasing mode with respect to the spectral maximum of the spontaneous emission. At the same time, the spectral shifts for the samples #1 and DCM-based samples #2 during the transitions from the spontaneous emission to random lasing are subtle (Figure 3 and Figure 4). Note that the effect of the spectral shift of the fluorescence maximum upon this transition manifests itself for some pumped fluorescent random media (see, e.g., [45]). However, a detailed analysis of the probable reasons for such a manifestation of the spectral shift for the R6G-based suspensions and its practical absence for other samples is beyond the scope of this work and is the subject of further research.

## 4. Theoretical Modeling

Theoretical consideration of the saturation effects of fluorescence spectral quality in over-threshold regimes of random lasing will be carried out taking into account two features of laser pumping conversion into fluorescence response of the pumped random media. The first feature relates to the granular structure of the pump field due to speckle modulation of laser radiation in the pumped random medium [26]. As a result, the fluorescent response is formed as a superposition of local contributions from laser-speckle-associated microscopic emitters, randomly distributed in the pumped volume. It can be expected that at high pump intensities the stochastic ensemble of local fluorescence emitters in the medium will tend to a certain saturated state (in particular, for the average population of the excited state of fluorophore molecules in the volumes of the emitters). The second feature is due to the absence of optical feedback between different local emitters in the ensemble. Accordingly, the purely stochastic nature of the amplification of partial components of the fluorescent field manifests itself due to stochastic acts of induced emission during random walks of the florescence photons in the medium until their exit.

### 4.1. Modeling of the Extreme Saturated States in Laser-Pumped Fluorescent Random Media

Previously [26], the effects of speckle modulation of pump laser radiation on the fluorescent response of a pumped random medium were considered. In the framework of this approach, we can describe the kinetics of the fluorescence response of a speckle-associated confined volume in a laser-pumped fluorescent medium using a system of coupled first-order differential equations:

(2) $$\begin{array}{l} \frac{df}{dt}=\frac{\sigma_{a}}{h\nu_{p}}I_{p}\left(t\right)\left\{1-f\right\}-\frac{{\langle \sigma_{st}\rangle }_{\lambda }}{{\langle h\nu_{f}\rangle }_{\lambda }}{\langle I_{f}\left(t\right)\rangle }_{\lambda }f+\\ +\frac{{\langle \sigma_{sa}\rangle }_{\lambda }}{{\langle h\nu_{f}\rangle }_{\lambda }}{\langle I_{f}\left(t\right)\rangle }_{\lambda }\left\{1-f\right\}-\delta f, \end{array}$$

(3) $$\begin{array}{l} \frac{d{\langle I_{f}\left(t\right)\rangle }_{\lambda }}{dt}=\{\frac{{\langle \sigma_{st}\rangle }_{\lambda }}{{\langle h\nu_{f}\rangle }_{\lambda }}{\langle I_{f}\left(t\right)\rangle }_{\lambda }f+\delta f-\frac{{\langle \sigma_{sa}\rangle }_{\lambda }}{{\langle h\nu_{f}\rangle }_{\lambda }}{\langle I_{f}\left(t\right)\rangle }_{\lambda }\left\{1-f\right\}-\\ -\frac{\sigma_{rad}\left(d_{em}\right)}{{\langle h\nu_{f}\rangle }_{\lambda }}{\langle I_{f}\left(t\right)\rangle }_{\lambda }\}n_{0}{\langle h\nu_{f}\rangle }_{\lambda }v. \end{array}$$

Equation (2) describes the evolution of an excited state population in an ensemble of fluorescence centers (fluorophore molecules) depending on the temporal dynamics of laser pumping $I_{p}\left(t\right)$. This evolution is considered in terms of the current value of the relative population $f\left(t\right)=n\left(t\right)/n_{0}$, where n(t) is the current volume concentration of the excited centers, and $n_{0}$ is the total concentration of centers in a pumped system. Equation (3) gives the current value of fluorescence intensity in a confined emitting volume with a characteristic size of $d_{em}$. In the case of laser pumping of strongly scattering random media, when the coherence length of pump radiation significantly exceeds the average propagation path of the laser light in the medium, the pump light field has a granular (speckled) structure. Accordingly, the value of $d_{em}$ is associated with the characteristic size of bulk laser speckles in the medium. It should be noted that, in the framework of the model of speckled pumping [26], the structural properties of the pumped system are characterized by only two parameters, such as $d_{em}$ and $n_{0}$. Also, the fluorescence response of the confined speckle-associated emitting volume is considered in terms of the wavelength-averaged current intensity ${\langle I_{f}\left(t\right)\rangle }_{\lambda }$; the averaging is carried out over the emission spectrum. Accordingly, the corresponding emission parameters are also wavelength-averaged; these parameters are the average energy of fluorescence photons ${\langle h\nu_{f}\rangle }_{\lambda }$, the cross section of stimulated emission ${\langle \sigma_{st}\rangle }_{\lambda }$, the self-absorption cross section ${\langle \sigma_{sa}\rangle }_{\lambda }$, and the rate of spontaneous emission δ. The parameters $\sigma_{a}$ and $h\nu_{p}$ relate to the absorption cross section of the fluorophore molecules and the photon energy of the pump radiation, respectively. The parameter v is the speed of light in the medium; it is applied to establish a relationship between the changes in the current population of excited states and the current fluorescence intensity in the emitting volume.

Specification of the model parameters $\sigma_{a},\;\delta ,\;{\langle h\nu_{f}\rangle }_{\lambda },$ and ${\langle \sigma_{st}\rangle }_{\lambda }$ for the studied R6G- and DCM-based systems was carried out using the results of preliminary experiments and datasets reported in the literature (see Table 2). In particular, a cross section of the stimulated emission can be obtained using the following expression [46,47]

(4) $$\sigma_{st}\left(\lambda ,I_{p}\right)=\frac{S\left(\lambda ,I_{p}\right)\lambda^{5}}{8\pi \tau_{s}cn_{ef}^{2}\int_{0}^{\infty }S\left(\lambda ,I_{p}\right)\lambda d\lambda }.$$

with further spectral averaging

(5) $${\langle \sigma_{st}\rangle }_{\lambda ,I_{p}}=\frac{\int_{0}^{\infty }\sigma_{st}\left(\lambda ,I_{p}\right)S\left(\lambda ,I_{p}\right)d\lambda }{\int_{0}^{\infty }S\left(\lambda ,I_{p}\right)d\lambda }.$$

**Figure 6:**
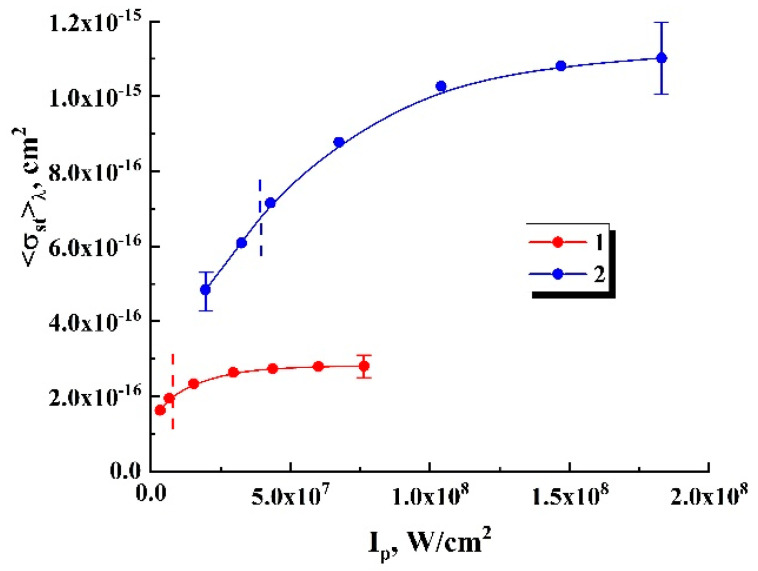
The wavelength-averaged cross sections of the stimulated emission ${\langle \sigma_{st}\rangle }_{\lambda }$ of fluorophore molecules against the pump intensity. 1—R6G molecules, 2—DCM molecules. Selectively shown error bars correspond to a confidence level of 0.9. Vertical dashed lines correspond to the random lasing thresholds for the samples #1.

Here, $\tau_{s}=\delta^{-1}$ is the characteristic time of the fluorescence decay. Figure 6 displays the recovered values of ${\langle \sigma_{st}\rangle }_{\lambda ,I_{p}}$ for the examined R6G- and DCM-based systems against the pump intensity. The recovery was carried out using Equations (4) and (5) on the basis of the experimentally obtained fluorescence emission spectra $S\left(\lambda ,I_{p}\right)$ at various intensities. The recovered datasets exhibit saturation of the spectrum-averaged cross sections ${\langle \sigma_{st}\rangle }_{\lambda ,I_{p}}$ with increasing pump intensity. In the further analysis, the saturated values of ${\langle \sigma_{st}\rangle }_{\lambda ,I_{p}}$ were used as model parameters for the system of Equations (2) and (3) (Table 2).

In the framework of the considered model of speckled pumping, the cross section of radiation losses $\sigma_{rad}$ of a confined emitting volume plays a crucial role in the radiative transfer between this emitting volume and the surrounding space; together with the current fluorescence intensity ${\langle I_{f}\left(t\right)\rangle }_{\lambda }$ in the speckle-associated local emitter, it characterizes the contribution of the given emitter to the observed integrated fluorescence output.

An upper estimate of the cross section of radiation losses for an isolated spherical emitter located in free space, when there is only a photon flux from the emitter to the outside, yields the value [26] $\sigma_{rad}^{\max}\approx 3/2n_{0}d_{em}$. Here, $d_{em}$ is the diameter of the emitter. This estimate is based on the following assumptions: at a given time, fluorescence photons are uniformly distributed over the volume of the emitter, and their possible directions of propagation are uniformly distributed in the solid angle of 4π. Accounting for the relationship between the flux of photons leaving the emitter and the corresponding negative contribution to the right side of Equation (6) leads to the above expression for the cross section of radiation losses in an isolated emitter. Radiation exchange between the neighboring local emitters in the pumped volume leads to a substantial decrease in the ensemble-averaged cross section $\langle \sigma_{rad}\rangle$ compared to the upper estimate: $\langle \sigma_{rad}\rangle <<\sigma_{rad}^{\max}$. This decrease can be taken into account by introducing the factor of radiation exchange $K_{rad}<<1$: $\langle \sigma_{rad}\rangle \approx K_{rad}/n_{0}\langle d_{em}\rangle$. In particular, the phenomenological model of radiation exchange in an ensemble of speckle-associated emitters uniformly distributed in a pumped multiple scattering medium [48] leads to the following expression for $K_{rad}$:

(6) $$K_{rad}\approx \frac{3}{2}\cdot \left\{1-\frac{1.07\pi {\langle d_{em}\rangle }^{2}l^{*}}{\Xi^{3}{\langle d_{sp}\rangle }^{3}}\exp\left(-\frac{1.15\langle d_{em}\rangle }{l^{*}}\right)\right\},$$

where $\langle d_{sp}\rangle$ is the characteristic size of the bulk laser speckles in the medium, and Ξ is the dimensionless structural parameter of the speckle field related to the ratio of the average distance between the neighboring speckles to $\langle d_{sp}\rangle$. Note that, in the framework of the considered model, the following relationship is considered: $\langle d_{em}\rangle \leq \langle d_{sp}\rangle <<l^{*}$.

**Figure 7:**
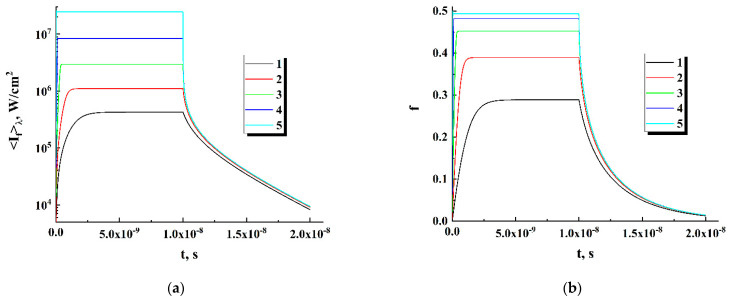
Saturation of ${\langle I_{f}\rangle }_{\lambda }$ (**a**) and f (**b**) during the action of a single laser pulse (modeling results). Pump intensity: 1 × 10^6^ W/cm^2^ (1); 3 × 10^6^ W/cm^2^ (2); 9 × 10^6^ W/cm^2^ (3); 2.7 × 10^7^ W/cm^2^ (4); 8.1 × 10^7^ W/cm^2^ (5).

Under appropriately high levels of pump intensity, the current values of the relative population f and fluorescence intensity ${\langle I_{f}\rangle }_{\lambda }$ in the local emitters (the left-hand sides of Equations (2) and (3), respectively) reach stationary levels over limited time intervals, which can be significantly shorter compared to the duration of pump laser pulses. In particular, Figure 7 displays the model behavior of f and ${\langle I_{f}\rangle }_{\lambda }$ when the local emitter is pumped by a rectangular laser pulse with a given value of $I_{p}$ around and above the threshold of random lasing. The absorption and emission parameters of the active medium in the emitter volume ($\sigma_{a}$,${\langle \sigma_{st}\rangle }_{\lambda }$, and δ) correspond to those of the R6G-doped samples (see Table 2), the pump pulse duration is set equal to 10 ns, and the concentration of R6G molecules in the medium is assumed to be equal to 2 × 10^18^ cm^−3^. The model value of the cross section of radiation losses $\sigma_{rad}$, in accordance with the results presented in [41], was chosen to be 1.4 × 10^−16^ cm^2^. It can be seen that stationary states of the local emitter for the pumping modes $I_{p}>I_{p,th}$ are reached during time intervals that are much shorter than the pump pulse duration. A similar tendency to a short-term transition to the stationary states of local emitters at $I_{p}>I_{p,th}$ is present in the case of simulated DCM-doped systems.

In the stationary state ($df/dt=0;\;d{\langle I_{f}\rangle }_{\lambda }/dt=0$), the system of the kinetic Equations (2) and (3) is reduced to the following form:

(7) $$\left\{ \begin{array}{l} \frac{\sigma_{a}}{h\nu_{p}}I_{p}\left(1-f\right)+\frac{{\langle \sigma_{sa}\rangle }_{\lambda }}{{\langle h\nu_{f}\rangle }_{\lambda }}{\langle I_{f}\rangle }_{\lambda }\left(1-f\right)-\frac{{\langle \sigma_{st}\rangle }_{\lambda }}{{\langle h\nu_{f}\rangle }_{\lambda }}{\langle I_{f}\rangle }_{\lambda }f-\delta f=0;\\ \frac{{\langle \sigma_{st}\rangle }_{\lambda }}{{\langle h\nu_{f}\rangle }_{\lambda }}{\langle I_{f}\rangle }_{\lambda }f+\delta f-\frac{\sigma_{rad}}{{\langle h\nu_{f}\rangle }_{\lambda }}{\langle I_{f}\rangle }_{\lambda }-\frac{{\langle \sigma_{sa}\rangle }_{\lambda }}{{\langle h\nu_{f}\rangle }_{\lambda }}{\langle I_{f}\rangle }_{\lambda }\left(1-f\right)=0; \end{array}\right.$$

In the case of the wavelength-averaged self-absorption cross section ${\langle \sigma_{sa}\rangle }_{\lambda }$ being small compared to other cross sections of the pumped medium, System (7) can be transformed into

(8) $$\left\{ \begin{array}{l} {\langle I_{f}\rangle }_{\lambda }=\frac{\delta f{\langle h\nu_{f}\rangle }_{\lambda }}{\sigma_{rad}-{\langle \sigma_{st}\rangle }_{\lambda }f};\\ {\langle I_{f}\rangle }_{\lambda }=\left(\frac{\sigma_{a}}{h\nu_{p}}I_{p}\left\{1-f\right\}-\delta f\right)\frac{{\langle h\nu_{f}\rangle }_{\lambda }}{{\langle \sigma_{st}\rangle }_{\lambda }f}. \end{array}\right.$$

**Figure 8:**
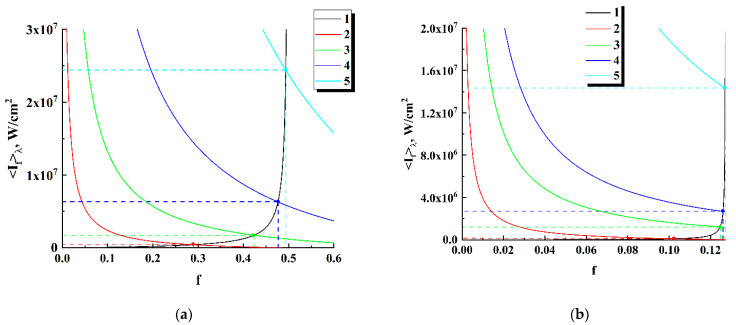
Graphical interpretation of the solution of System (11). Curve (1) corresponds to the first equation; curves (2–5) correspond to the second equation at various values of the pump intensity. Horizontal and vertical dashed lines mark unique solutions of (11) for the given pump intensities. (**a**) R6G-filled local emitter, $I_{p}=$ 1 × 10^6^ W/cm^2^ (2), $I_{p}=$ 5 × 10^6^ W/cm^2^ (3), $I_{p}=$ 2 × 10^7^ W/cm^2^ (4), $I_{p}=$ 8 × 10^7^ W/cm^2^ (5); (**b**) DCM-filled local emitter, $I_{p}=$ 1 × 10^7^ W/cm^2^ (2), $I_{p}=$ 5 × 10^7^ W/cm^2^ (3), $I_{p}=$ 1 × 10^8^ W/cm^2^ (4), and $I_{p}=$ 5 × 10^8^ W/cm^2^ (5).

System (8) has a unique solution $\left(f,{\langle I_{f}\rangle }_{\lambda }\right)$ for a given set of emitter parameters ($\sigma_{a}$, δ, ${\langle \sigma_{st}\rangle }_{\lambda }$, $\sigma_{rad}$, $h\nu_{p}$, and ${\langle h\nu_{f}\rangle }_{\lambda }$) at a used pump intensity $I_{p}$; graphical interpretation of the solution procedure is illustrated by Figure 8. As in the case of modeling the time dependences of ${\langle I_{f}\left(t\right)\rangle }_{\lambda }$ and f(t) (Figure 7), the cross section $\sigma_{rad}$ of the radiation losses of the local emitters is set equal to ≈1.4⋅10^−16^ cm^2^. It can be seen that, with an increase in the pump intensity, the relative population of excited level f gradually approaches the extreme value $f_{ext}=\sigma_{rad}/{\langle \sigma_{st}\rangle }_{\lambda }$; accordingly, when $f\to f_{ext}$, the fluorescence intensity ${\langle I_{f}\rangle }_{\lambda }$ linearly rises depending on $I_{p}$. Indeed, at high pump intensities, when $\left(\sigma_{a}/h\nu_{p}\right)I_{p}\left(1-f\right)>>\delta f$, Equation (2) of System (8) tends to the following form: ${\langle I_{f}\rangle }_{\lambda }\approx \left\{\left(\sigma_{a}/{\langle \sigma_{st}\rangle }_{\lambda }\right)\cdot \left({\langle h\nu_{f}\rangle }_{\lambda }/h\nu_{p}\right)\cdot \left({\langle \sigma_{st}\rangle }_{\lambda }-\sigma_{rad}\right)/\sigma_{rad}\right\}I_{p}$. Note that the DCM-filled local emitter exhibits a significantly lower value for the relative population approaching the saturated state compared to the R6G-filled emitter (Figure 8a against Figure 8b) due to the significantly larger cross section of stimulated emission of DCM molecules (Table 2).

### 4.2. A Probabilistic Model for the Spectral Narrowing of the Fluorescence Output in Pumped Random Media

**Figure 9:**
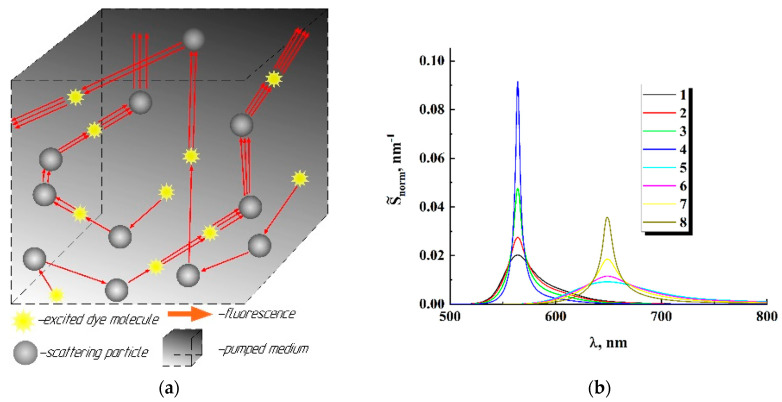
(**a**) Illustration of the considered probabilistic model; (**b**) modeled normalized spectra of fluorescence output of R6G-doped (1–4) and DCM-doped (5–8) pumped randomly inhomogeneous layers with $l^{*}=$ 4 µm; (1, 5) − $l_{st}=$ 1000 µm; (2, 6) − $l_{st}=$ 250 µm; (3, 7) − $l_{st}=$ 62.5 µm; (4, 8) − $l_{st}=$ 15.63 µm.

In consideration of the spectral narrowing of the fluorescence response in random media with increasing pump intensity, we use the general concepts of the discrete scattering model of radiation propagation in the medium (see, e.g., [25]). In the framework of these concepts, a multiple scattered light field in a random medium can be considered as a superposition of non-correlated partial components (waves) propagating in the medium along statistically independent random paths. In our case, let us consider an arbitrary act of spontaneous emission of an excited fluorophore molecule in a laser-pumped fluorescent medium. A photon emitted with an energy of hc/λ randomly travels in the medium before leaving it (here, λ is the wavelength of emission in the vacuum). Traveling along the propagation path in the pumped medium, it can interact with an excited fluorophore molecule and induce an act of stimulated emission. The n-fold sequential repetition of these interactions along the trace will result in the exit of an n+1-fold photon packet from the medium (Figure 9a). Further details of the derivation of the formula for the normalized spectral density of the fluorescent response of a pumped randomly inhomogeneous medium are presented in the Supplementary Materials to this article.

This spectral density is obtained (see the Supplementary Materials) as

(9) $$\tilde{S}\left(\lambda \right)=\int_{0}^{\infty }{\tilde{S}}_{\frac{s}{l_{st}}+1}\left(\lambda \right)\;\rho \left(s\right)ds.$$

Here ${\tilde{S}}_{s/l_{st}+1}\left(\lambda \right)$ is the modified spectral density of spontaneous emission, and ρ(s) is the probability density function of the propagation paths s of photon packets in a pumped medium. Modification of the normalized spectral density $S_{sp}\left(\lambda \right)$ of spontaneous fluorescence emission is carried out in the following way (see Equation (S8) in the Supplementary Materials)

(10) $${\tilde{S}}_{n+1}\left(\lambda \right)=\left(\frac{1}{\lambda }\right)\cdot \frac{{\left\{\lambda S_{sp}\left(\lambda \right)\right\}}^{n+1}}{\int_{0}^{\infty }{\left\{\lambda^{\prime }S_{sp}\left(\lambda^{\prime }\right)\right\}}^{n+1}d\lambda^{\prime }},$$

where the index n denotes the number of successful acts of stimulated emission during the propagation of a photon packet in a medium. This number can be defined as $n\approx s/l_{st}$, where $l_{st}\approx {\left({\langle \sigma_{st}\rangle }_{\lambda }n_{0}\langle f\rangle \right)}^{-1}$ is the characteristic propagation scale of the stimulated emission in the medium, and 〈f〉 is the averaged population of the exited state of fluorophore molecules over an ensemble of local fluorescence emitters in the pumped medium.

In the examined random nanostructured media, spatial distributions of the pumping and fluorescent radiation (and, correspondingly, the pathlength statistics of the outgoing fluorescence) are controlled by the optical transport parameters of the pumped medium and illumination conditions. The optical transport parameters in the case of a near-isotropic scattering typical for nanostructured systems are effective refractive indices $n_{eff}$, transport mean free paths $l^{*}$, and absorption lengths $l_{a}$ as reciprocals of the absorption coefficients at the corresponding wavelengths. A Monte Carlo simulation was applied to mimic the pathlength distributions ρ(s) of the outgoing fluorescence depending on $l^{*}$, $n_{eff}$, $l_{a}$, and illumination conditions in the case of a slab geometry of the pumped medium. In the modeling, the slab thickness L significantly exceeded the transport mean free path and absorption length; this typically corresponded to the applied experimental conditions of fluorescence excitation in the examined systems (except in the limited case of low volume fractions of anatase nanoparticles in the samples #2). At the first stage of the procedure, stationary spatial distributions of excited fluorophore molecules in the pumped medium were simulated using the tracing of randomly walking individual pumping photons until their absorption. The coordinates of the absorption points (i.e., the excited fluorophore molecules) were recorded for further application at the second stage of the simulation. During the simulation procedure, the number of pump photons coming into the medium through the upper boundary was chosen to be 10^7^. The collected data were used as the dataset for the initial points of the fluorescent photons at the second stage of simulation. Starting from these points, randomly walking fluorescence photons (the partial contributions to the fluorescence output) emerging through the upper boundary were traced in the medium until they entered the free space. After finishing each simulation run, the accumulated set {s} of possible propagation paths of fluorescence radiation in the medium was subjected to a frequency counting procedure. As a result, for each given value of $l^{*}$, a sample probability density function ρ(s) was obtained, which was used to recover the fluorescence emission spectrum $\tilde{S}\left(\lambda \right)$ according to Formulas (9) and (10) at a given value of $l_{st}$. As an example, Figure 9b displays the results of such a recovery procedure for R6G- and DCM-doped thick layers of an isotropically scattering random medium with $l^{*}=$ 4 µm; the normalized emission spectra ${\tilde{S}}_{norm}\left(\lambda \right)=\tilde{S}\left(\lambda \right)/\int_{0}^{\infty }\tilde{S}\left(\lambda \right)d\lambda$ are plotted for various values of the stimulated emission length $l_{st}$, from 1000 µm (close-to-spontaneous emission) to 16.25 µm (expressed narrowing of the emission spectrum).

The modeled data in Figure 9b can be compared to the experimentally obtained normalized emission spectra of the samples #1 at various pump levels (Figure 2); note that the value of the mean transport free path used for the modeled systems is close to the values of $l^{*}$ for the examined samples #1. Obviously, an increase in the pump intensity should lead to a decrease in $l_{st}$ due to a rising stationary population of an excited state of the dye molecules. On the other hand, this rise is limited by the above-established condition $f_{ext}=\sigma_{rad}/{\langle \sigma_{st}\rangle }_{\lambda }$ at high pump intensities, and therefore we should expect certain limitations in the spectral output of the pumped medium. This point is discussed in detail in the following section.

## 5. Discussion

**Figure 10:**
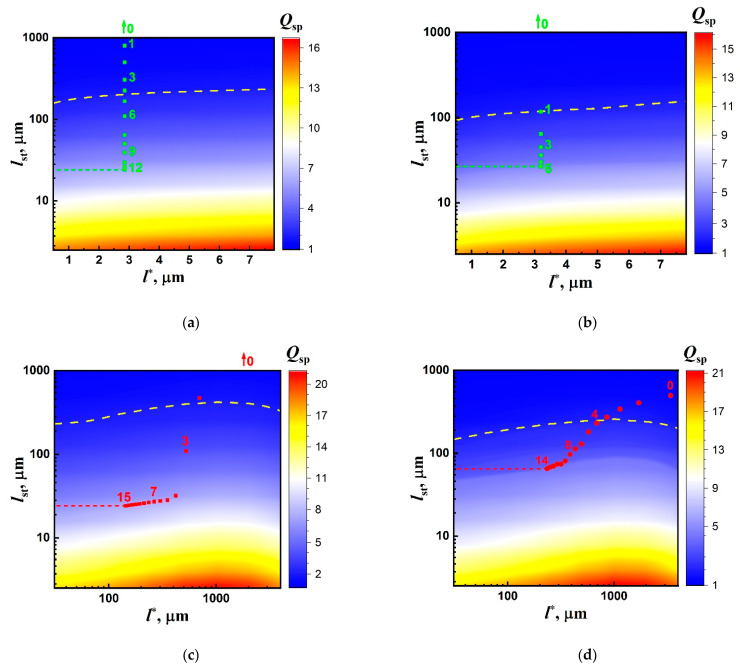
2D color maps of the $Q_{sp}$ states of the examined systems in the $\left(l^{*},l_{st}\right)$ domain (modeling results) with the experimental data (separate cyan and red markers; selective numbering of the markers corresponds to those used in Figure 3 and Figure 5). (**a**)—R6G-doped layer (#1); (**b**)—DCM-doped layer (#1); (**c**)—R6G-based suspension (#2); (**d**)—DCM-based suspension (#2). Dashed cyan and red lines mark the expected values $l_{st,ext}$ corresponding to the crowded experimental data.

Figure 10 shows a set of color maps displaying model distributions of the enhancement factor $Q_{sp}$ in the $\left(l^{*},l_{st}\right)$ domain together with the empirical values of $Q_{sp}$ for the examined samples #1, 2. The model values of $Q_{sp}$ for color mapping were obtained from a set of recovered spectra ${\tilde{S}}_{norm}\left(\lambda \right)$ generated using the above-described Monte Carlo modeling and recovery procedure. The $l^{*}$ and l values for the modeled R6G- and DCM-doped random layers with isotropic scattering varied in wide intervals to cover the experimentally observed ranges of $Q_{sp}$ for the examined densely packed layers (#1) and suspensions (#2) of anatase nanoparticles. Accordingly, for the first case, detailed color maps with high resolution and a narrow range along the $l^{*}$-axis are used (Figure 10a,b). The $Q_{sp}$ states for the pumped samples #2 are presented on the “panoramic” color maps with significantly wider displayed intervals in the $l^{*}$ domain (Figure 10c,d).

The selectively numbered markers corresponding to the empirical data (Figure 3 and Figure 5) were plotted on color maps based on the corresponding values of $Q_{sp}$ and $l^{*}$. In the case of samples #1 (the densely packed layers), assuming that the mean transport free path is independent of the pump intensity, the markers for these data should be located on the vertical lines corresponding to the $l^{*}$ values for the examined systems at the average wavelengths of the fluorescence outputs (see Table 1). In contrast, the used experimental conditions for samples #2 (constant pump intensity at the boundaries of the samples and a varying mean transport free path of radiation propagation) should lead to the arrangement of markers predominantly in the $l^{*}$ direction. An exception involves cases of large values of $l^{*}$ at low volume fractions of anatase nanoparticles, when the multiple scattering mode in the samples is not yet realized. On all color maps, dashed yellow lines correspond to the condition of the random lasing threshold ($Q_{sp}=$ 2).

The behavior of the empirical data (crowding of marker positions at high pump intensities $I_{p}>I_{p,th}$ for the samples #1 and their arrangement along the isolines $Q_{sp}=const$ for the samples #2 at appropriately small values of $l^{*}$) clearly indicates the saturation effect for the characteristic scale $l_{st}$. In the case of over-pumped samples (Figure 10a–c), such saturation can be considered in terms of reaching the extreme population of the excited state of fluorophore molecules in local emitters $f_{ext}=\sigma_{rad}/{\langle \sigma_{st}\rangle }_{\lambda }$. In this case, the stimulated emission length $l_{st}\approx {\left({\langle \sigma_{st}\rangle }_{\lambda }n_{0}\langle f\rangle \right)}^{-1}$ tends to $l_{st,ext}\approx {\left(\sigma_{rad}n_{0}\right)}^{-1}$. On the other hand, $\sigma_{rad}\approx K_{rad}/n_{0}\langle d_{em}\rangle$ and, accordingly, $l_{st,ext}\approx \langle d_{em}\rangle /K_{rad}$. Thus, the extreme value of $l_{st}$ for the over-pumped samples does not depend on the concentration and emission properties of the applied active medium (fluorescent dye). This parameter is determined only by the characteristic size of speckle-associated local emitters $\langle d_{em}\rangle$ and the factor $K_{rad}$ of radiation exchange between the emitters. In the diffusion mode of the laser light transfer in the pumped media, the expected size of bulk laser speckles in the medium is of the order of $\lambda_{p}/\Omega$, where Ω is the half-width of the angular spectrum of diffusing laser light in the medium.

As follows from Figure 10a–c, the estimated values of $l_{st,ext}$ are between 20 µm and 30 µm ($l_{st,ext}^{\#1,R6G}\approx$ 25 µm; $l_{st,ext}^{\#1,DCM}\approx$ 28 µm; $l_{st,ext}^{\#2,R6G}\approx$ 25 µm). On the other hand, the previously obtained estimated values of $\langle d_{em}\rangle$ for 532 nm-pumped dense random media [48] are between 70 nm and 100 nm; the corresponding values of $K_{rad}$ are between 0.002 and 0.004. Accordingly, the extreme value of $l_{st}$ in the case of green laser pumping is expected to be between 17 µm and 50 µm. This estimate satisfactorily agrees with the above-presented $l_{st,ext}$ values for the examined systems under the condition of over-pumping. At the same time, the applied pumping mode is not sufficient to achieve the extreme maximal values of ${\langle \sigma_{st}\rangle }_{\lambda }$ (Figure 6, curve 2) and 〈f〉 (Figure 8b) for the DCM-based suspension (Figure 10d). The corresponding saturated value of $l_{st}\approx$ 65 µm is approximately 2.5 times larger than $l_{st,ext}$ for other samples.

A remarkable feature is that the stimulated emission length $l_{st}$ even in the saturated states is many times greater than the characteristic size of speckle-associated local emitters of fluorescence. Accordingly, the probability of formation of a photon packet consisting of two or more photons in the volume of the emitter after the act of spontaneous emission in it is vanishingly small. Thus, the factor of radiation exchange between emitters plays an important role in the formation of the stimulated component of the fluorescent response of the pumped medium.

It should be noted that, despite the significant difference in the achievable minimal values of $l_{st}$ for R6G- and DCM-based samples #2 at the applied pump level $I_{p}\approx$ 8.8⋅10^7^ W/cm^2^ (≈25 µm against ≈65 µm), the effect of $l_{st}$ saturation occurs for both samples at $l^{*}\leq$ 600 ÷700 µm. For these conditions, the ratios of the scattering layer (cuvette) thickness to the TMFP values exceed ≈7 ÷ 8, and fluorescence transfer in the pumped layers has a close-to-diffusion character. In particular, the question regarding the geometric constraints of light diffusion in multiple scattering media with slab geometry was previously considered in [49], and the established $L/l^{*}$ criterion of transition from the low-step-scattering to the diffusion mode of light transport in multiple scattering slabs is close to the mentioned value. In this case, a stochastic ensemble of bulk laser speckles with a characteristic size of the order of $\lambda_{p}/\Omega$ is formed in the pumped medium.

## 6. Conclusions

Thus, the obtained results allow us to conclude that the fundamental limitation of the spectral quality of the fluorescent response observed in random lasing experiments at high pump intensities can be interpreted in terms of reaching the extreme saturation of the excited state population in an ensemble of speckle-associated local fluorescence emitters. It is evident from theoretical consideration that saturation of the excited state of fluorophore molecules in speckle-associated local emitters occurs when the flux of photons flowing out from the emitter volume and the flux of photons generated in the volume become close to each other. In other words, competition between these processes leads to the establishment of a dynamic equilibrium in the ensemble of local emitters at a given level of pump intensity. Accordingly, the extreme population of the excited state in local emitters is determined only by the ratio of their average cross section of radiation losses and cross section of stimulated emission of fluorophore molecules.

As saturation of the excited state population is near, the stimulated emission length (or, in other words, the amplification length for the stimulated emission) in the medium tends to the extreme minimal value. This minimal value does not depend on the emission properties of the fluorophore and properties of the scattering matrix but is determined by the characteristic size of local emitters in the medium and the radiation exchange factor. This conclusion is supported by the presented experimental data on random lasing excitation in nanostructured fluorescent media with substantially various emission characteristics and morphology, as well as the results of statistical modeling of fluorescence transfer and amplification in the examined systems using the considered probabilistic model. Note that for various nanostructured highly scattering media characterized by a diffuse propagation mode of pump radiation with a given wavelength, the characteristic sizes of speckles in the pump fields are expected to be close to each other. This follows from the relationship between the wavelength of laser radiation, characteristic size of bulk laser speckles in the medium, and width of the angular spectrum of multiply scattered radiation in the medium, which is practically isotropic for such systems. Accordingly, the extreme amplification lengths of fluorescence in such systems must differ insignificantly regardless of the scattering matrices and fluorophores. Thus, this makes it possible to explain the close values of the enhancement factors of the fluorescence spectral quality at high pump intensities observed in numerous random lasing experiments with various systems of the “scattering matrix–laser dye” type.

The discussed concept of fluorescence excitation in a random ensemble of uncorrelated local emitters under external laser pumping belongs to the class of discrete models of radiative transfer. At the same time, as mentioned above, when describing fluorescence transfer and amplification in pumped randomly inhomogeneous media, some researchers resort to the diffusion approximation of the radiative transfer theory (“the continuity concept”), which does not take into account the local granular structure of the pump field (and, accordingly, the fluorescence response). It seems useful to “build a bridge” between these two approaches in terms of establishing a relationship between the parameters of an ensemble of local emitters and parameters of a pumped random medium introduced in the framework of diffusion approximation. These parameters are, on the one hand, the average cross section of radiation losses of local emitters, the factor of radiation exchange, etc., and the light diffusion coefficient, the reduced scattering coefficient, etc., on the other hand. This point is considered as a topic for a further study.

Finally, we hope that the findings presented in this work could be useful for further development of methods for fluorescent diagnostics of dispersive nanosystems and composite nanomaterials.

## Figures and Tables

**Table 1:** Estimated values of $l^{*}$ and $n_{eff}$ for the used scattering matrices at pump and emission wavelengths.

Type of the Samples	Pump Wavelength, 532 nm	Average Wavelength 1 of Emission, ≈597 nm	Average Wavelength 2 of Emission, ≈675 nm
Samples #1 (dense layers)	$n_{ef}\approx$ (1.54 ± 0.03) ^1^	$n_{ef}\approx$ (1.52 ± 0.03) ^1^	$n_{ef}\approx$ (1.50 ± 0.03) ^1^
	$l^{*}\approx$ (2.50 ± 0.20) µm	$l^{*}\approx$ (2.85 ± 0.20) µm	$l^{*}\approx$ (3.20 ± 0.20) µm
Samples #2 (suspensions)	$n_{ef}\approx$ 1.36 ^2^	$n_{ef}\approx$ 1.36 ^2^	$n_{ef}\approx$ 1.36 ^2^
	$l^{*}\approx$ (3.2 µm)/ρ	$l^{*}\approx$ (5.07 µm)/ρ	$l^{*}\approx$ (8.3 µm)/ρ

^1^ Estimated using the effective medium theory (CPA approach [38,39,40]). ^2^ taken from [44].

**Table 2:** Absorption and emission parameters of the used fluorescent components.

**Fluorophore**	${\langle h\nu_{f}\rangle }_{\lambda }\times 10^{19},\;J$	$\sigma_{a},\;\times 10^{18},\;{\mathrm{cm}}^{2}$	${\langle \sigma_{st}\rangle }_{\lambda }\times 10^{16},\;{\mathrm{cm}}^{2}$	$\delta \times 10^{-8},s^{-1}$
**R6G Solution**	≈3.33	≈92.0 ^1^	2.8 ^2^	2.56 ^3^
**DCM Solution**	≈2.94	≈6.1 ^1^	11.0 ^2^	10.0 ^3^

^1^ Estimated using measurements of the absorption coefficients of R6G and DCM solutions; ^2^ Correspond to the expected saturated values (see Figure 6); ^3^ Estimated as $\delta =1/\tau_{s}$ using the reported data on $\tau_{s}$ for R6G [30,31,32] and DCM [33].

## Data Availability

Not applicable.

## Supplementary Materials

The following supporting information can be downloaded at: https://www.mdpi.com/article/10.3390/nano12142450/s1, Section S1: Details of the Sample Preparation and Characterization; Section S2: Derivation of the Relationship for the Spectral Density of the Fluorescence Response in the Framework of a Probabilistic Model; references [25,26,34,35,36,37,38,39,40,41,42,43] are also cited in the Supplenentary Materials.
