# Supplementary material for: Saturated Emission States in Fluorescent Nanostructured Media: The Role of Competition between the Stimulated Emission and Radiation Losses in the Local Emitters of Fluorescence
Source: Nanomaterials (Basel). 2022 Jul 17;12(14):2450. doi: 10.3390/nano12142450 (PMC9315858; doi:10.3390/nano12142450)
Supplement: Supplementary file 1 [file nanomaterials-12-02450-s001.zip › nanomaterials-1822505-supplementary.pdf]

nanomaterials

Supplementary Materials to "Saturated Emission States in Fluorescent Nanostructured Media: the Role of Competition between the Stimulated Emission and Radiation Losses in the Local Emitters of Fluorescence"

Dmitry Zimnyakov [1,2,*], Sergey Volchkov [1], Leonid Kochkurov [1] and Alexander Dorogov [1]

1 Yury Gagarin State Technical University of Saratov, Physics Department, 410054 Saratov, Russian Federation; zimnykov@mail.ru (D.Z.); volchkov93@bk.ru (S.V); lkochkurov@gmail.com (L.K.); sanek_9993@mail.ru (A.D.)
2 Precision Mechanics and Control Institute of Russian Academy of Sciences, 24 Rabochaya st., 410024, Saratov, Russian Federation; zimnykov@mail.ru (D.Z.)
* Correspondence: zimnykov@mail.ru; Tel.: +7-845-299-8624 (D.Z.)

S1. Details of the Sample Preparation and Characterization

During the preparation of samples of the first type, the weighed portions of anatase nanopowder (approximately 190 mg) were placed in open cylindrical containers, leveled in thickness, and slightly pressed. The containers were metal rings with an inner diameter of 20 mm and the height of 1 mm, fixed on glass substrates. The use of glass substrates as the bottom walls of containers made it possible to characterize the samples by measuring their diffuse transmission (see below). After filling the containers, leveling the thickness and compacting the powder layers, they were carefully soaked with dye solutions. The required volumes of solutions were roughly estimated based on the value of the filled volume of the container and expected volume fraction of anatase particles in the samples. The thickness of the prepared samples was measured using the OCT system (OSC1300SS, the product of Thorlabs Co., Newton, NJ, USA) and was ≈ 1.08 mm; thickness variations over the sample surface did not exceed 5%. Accordingly, the volume fraction of anatase nanoparticles in the layers was estimated as $\rho \approx 0.22 \pm 0.02$.

In preparation of the second group of samples, various portions of anatase nanopowder (from 20 mg to 800 mg) were added to dye solutions (4 ml) and thoroughly mixed. The samples were put in 5 mm-thick glass cuvettes. To minimize the aggregation of nanoparticles, they were sonified in an ultrasonic bath for 20 minutes before measurements.

Among other factors, the average dwell times (and, accordingly, the average propagation paths) of pump and fluorescence photons in the examined systems play a key role in nonlinear conversion of pump radiation into fluorescence response of the systems. In addition to geometric factors (sample thickness, size of irradiated area, etc.), the average propagation paths are determined by optical transport parameters of the irradiated medium. Therefore, before spectral measurements, the samples were characterized by the values of optical transport parameters for the laser pump wavelength (532 nm) and average wavelengths in the dye fluorescence bands using the measured data relating the corresponding collimated and diffuse transmission coefficients.

In terms of the radiative transfer theory, light transport in random media is controlled by the following set of optical parameters: the absorption coefficient μ_a, the scattering coefficient μ_s, and the reduced scattering coefficient μ_s' [S.1]. The scattering and reduced scattering coefficients are related to each other as $\mu_s' = (1-g)\mu_s$, where g is the scattering anisotropy parameter. In our case, for subsequent analysis, it is more convenient to use the characteristic scales of radiation propagation, which are reciprocals of μ_a, μ_s, and μ_s': the absorption length $l_a = \mu_a^{-1}$, the mean scattering free path (MSFP) $l = \mu_s^{-1}$, and the mean transport free path (MTFP) $l^* = (\mu_s')^{-1} = l/(1-g)$ (see, e.g., [S.2]). It should

be noted that the studied anatase-based scattering matrices are characterized by the negligible absorption ($l_a \to \infty$) at the wavelengths of laser pumping (532 nm) and fluorescence outputs of the doping fluorophores (between 550 nm and 800 nm). In addition, these nanostructured matrices are actually random ensembles of Rayleigh scatterers with close-to-isotropic scattering ($0 < g << 1$). Accordingly, in such systems, multiple light scattering in the absence of the dye component can be fairly considered in terms of only the MTFP value in combination with an effective refractive index of the medium n_{ef}. This approach was previously justified in [S.3].

From this point of view, the measured diffuse transmission coefficients $T_d(\lambda)$ for the samples of the first group were used to recover the corresponding MTFP values $l^*(\lambda)$. The recovery procedure was based on a well-known relationship between T_d and l^* for a layer of a multiply scattering medium (see. e.g., [S.4, S.5]):

$$T_d(\lambda) = \frac{\left[1 + K_1(\lambda)\right] l^*(\lambda)}{L + \left[K_1(\lambda) + K_2(\lambda)\right] l^*(\lambda)} \quad \text{(S1)}$$

where $K_1(\lambda) = K_1\left[n_{eff}(\lambda)\right], K_2(\lambda) = K_2\left[n_{eff}(\lambda)\right]$ are dimensionless coefficients determined by the values of an integral reflectivity of the layer boundaries [S.6]. The applied procedure of $l^*(\lambda)$ recovery from the measured values of $T_d(\lambda)$ (including evaluation of $n_{eff}(\lambda)$ using a coherent potential approximation of an effective medium theory [S.7, S.8] and, on this basis, estimation of $K_1\left[n_{eff}(\lambda)\right], K_2\left[n_{eff}(\lambda)\right]$) is similar to that described in [S.9].

The diffuse transmittance coefficients were measured for the dye-free samples of the first group using a specially designed setup. This setup consisted of a broadband light source (the DH-2000BAL product of the Ocean Optics Co., Dunedin, FL, USA), an integrating sphere 2P4 from the the ThorLabs Co. (Newton, NJ, USA), and a spectrometer (QE65000 from the Ocean Optics Co.). The spectrometer was connected with the integrating sphere via a fiber-optic patch-cord (P200-2-UV-VIS type from the Ocean Optics Co. Dunedin, FL, USA). The measured layers of anatase nanoparticles in the containers totally overlapped the input port of the integrating sphere; the layers were impregnated by amounts of solvent (ethanol) without the dye equal to the amounts of dye solutions used for further spectral measurements.

The satellite samples of the second group (dye-free suspensions of anatase nanoparticles in the solvents) were characterized using the measurements of the collimated transmittance in 5-mm-thick cuvettes at the pump and fluorescence wavelengths. In the case of close-to-isotropic scattering, attenuation of the transmitted collimated beam can be described in terms of the generalized Bouguer's law:

$$T_c(\lambda) \approx \exp\left[-\frac{L_c}{l^*(\lambda)}\right] \quad \text{(S2)}$$

where $T_c(\lambda)$ is the collimated transmittance coefficient and L_c is the cuvette thickness. The measurements of $T_c(\lambda)$ were carried out using the above mentioned broadband light source DH-2000BAL in combination with a cuvette holder (the CUV-UV product from the Ocean Optics Co.) with incorporated collimators (the 74UV products from the Ocean Optics Co. Dunedin, FL, USA) and the QE65000 spectrometer. All the units were connected with each other using fiber-optic patch-cords of P200-2-UV-VIS type. After careful calibration made to take into account Fresnel reflections at the interfaces, the collimated transmittance of the suspensions was measured for the pump wavelength and average fluorescence wavelength as the function of the volume fraction of anatase nanoparticles in the suspensions. Based on the obtained data on $T_c(\lambda)$ at the volume fractions

of anatase nanoparticles (in the range from ≈ 0.0013 to ≈ 0.049), the ratios between the volume fractions of nanoparticles and the MTFP values of the prepared suspensions were established. The obtained results for the prepared scattering matrices are summarized in Table 1; values $\bar{\lambda}_1$ and $\bar{\lambda}_2$ correspond to the average wavelengths of emission for the fluorescent components used in the experiments (see below). These average wavelengths were calculated as $\bar{\lambda}_{1,2} = \int_0^\infty \lambda S_{sp1,2}(\lambda) d\lambda \Big/ \int_0^\infty S_{sp1,2}(\lambda) d\lambda$, where $S_{sp1,2}(\lambda)$ are the corresponding emission spectra of R6G and DCM at low pump intensities.

Assuming the spheroidal shape of the scattering sites in the prepared suspensions, the following relationship between the scattering efficiency factor Q_{sca} [S.1, S.10], the average size of the scattering site $\langle d_s \rangle$, the volume fraction of the sites ρ, their scattering cross-section σ_s, and the MSFP and MTFP values of the suspension can be established within the weak scattering limit [S.11]:

$$l^* \approx l = \left(\langle \sigma_s \rangle n_p\right)^{-1} = \left\{ \frac{Q_{sca}\pi\langle d_s \rangle^2}{4} \cdot \frac{6\rho}{\pi\langle d_s \rangle^3} \right\}^{-1} = \frac{2\langle d_s \rangle}{3Q_{sca}\rho}, \quad \text{(S3)}$$

where n_p is particle concentrations in the suspensions. The estimates of the ratios $\left(\langle d_s \rangle / Q_{sca}\right)_{\lambda_p}$, $\left(\langle d_s \rangle / Q_{sca}\right)_{\bar{\lambda}_1}$, and $\left(\langle d_s \rangle / Q_{sca}\right)_{\bar{\lambda}_2}$ for the prepared suspensions in the range of ρ from 0.0013 to 0.049 gave the values ≈ 4.8 μm, ≈ 7.6 μm, and ≈ 12.5 μm, respectively. Being compared to the theoretical dependence of d/Q_{sca} on d for the anatase spherical particles, which was obtained using the online scattering Mie calculator [S.12], we arrived to the following estimate for $\langle d_s \rangle$ in the prepared suspensions: $\langle d_s \rangle \approx$ (45 ± 5) nm. Thus, it can be concluded that the role of particle aggregation in the sonicated suspensions is relatively small ($\langle d_s \rangle$ exceeds the average particle size specified for the raw product # 637254 by no more than two times) and the assumption relating the close-to-isotropic scattering mode of the pumping and fluorescence radiation in the prepared suspensions is valid. In estimating the MTFP values for the volume fractions up to 0.049, we used the inverse linear relationship $l^* \sim 1/\rho$; this assumption corresponds to the case of a weak scattering limit, when interactions of the local scattered light fields from the neighboring scatterers are negligible. The results of numerous studies of this issue (see, e.g., [S.11]) allow us to conclude that cooperative effects in multiple scattering become significant (and, accordingly, the inverse linear dependence $l^* \sim 1/\rho$ fails) at the volume fractions substantially exceeding the maximal value of 0.049 used in our experiments. The refractive indices for the samples # 2 were taken equal to those of the solvent (ethanol) because of small ρ values of anatase nanoparticles.

S2. Derivation of the Relationship for the Spectral Density of the Fluorescence Response in the Framework of a Probabilistic Model

Because of statistical independence of the stimulated emission events and randomness of the medium, conditional probability to detect this $n+1$-fold photon packet at the wavelength of λ can be expressed as

$$\tilde{p}_{n+1}(\lambda) = p_{sp}(\lambda)\, p_{st}^n(\lambda), \quad \text{(S4)}$$

where $\tilde{p}_{n+1}(\lambda)$ is conditional probability of finding the $n+1$-fold photon packet at the wavelength of λ in the ensemble of outgoing fluorescence photons, $p_{sp}(\lambda)$ and $p_{st}(\lambda)$ are absolute probabilities of a spontaneous and stimulated emission of fluorescence photons at the corresponding wavelength.

The number of stimulated emission acts along the propagation trace with the length of s can be approximately evaluated as $n \approx s/l_{st}$, where l_{st} is a characteristic scale of the stimulated emission in the medium. In turn, the value of l_{st} can be estimated as $l_{st} \approx \left(\langle \sigma_{st} \rangle_{\lambda} n_0 \langle f \rangle\right)^{-1}$ (here, $\langle f \rangle$ is the averaged population of the exited state of fluorophore molecules over an ensemble of local fluorescence emitters in the pumped medium). An absolute probability to find the $n+1$-fold photon packet can be obtained using the following normalization condition

$$p_{n+1}(\lambda) = \frac{\tilde{p}_{n+1}(\lambda)}{\int_0^\infty \tilde{p}_{n+1}(\lambda) d\lambda}. \quad \text{(S5)}$$

In the stationary emission mode, the single-act emission probabilities $p_{sp}(\lambda)$ and $p_{st}(\lambda)$ can be connected with the spectral density $S_{sp}(\lambda) = S(\lambda, I_p \to 0)$ of a spontaneous fluorescence via the following relationship:

$$p_{sp}(\lambda) = \frac{\left(S_{sp}(\lambda)/(hc/\lambda)\right)}{\int_0^\infty \left(S_{sp}(\lambda)/(hc/\lambda)\right) d\lambda} = \frac{\lambda S_{sp}(\lambda)}{\int_0^\infty \lambda S_{sp}(\lambda) d\lambda}, \quad p_{sp}(\lambda) \approx K p_{st}(\lambda). \quad \text{(S6)}$$

Here, we assume that the probability for a travelling photon packet with the wavelength of λ to interact with an excited fluorophore molecule, which is ready to emit an additional photon in the stimulated emission act is proportionally related to the probability of a spontaneous emission at this wavelength (the proportionality coefficient is denoted as K). Under this assumption, the expression for the absolute probability $p_{n+1}(\lambda)$ is reduced to the following form (note that the proportionality coefficient K is excluded from a further consideration during normalization procedure):

$$p_{n+1}(\lambda) = \frac{\left\{\lambda S_{sp}(\lambda) \Big/ \int_0^\infty \lambda S_{sp}(\lambda) d\lambda\right\}^{n+1}}{\int_0^\infty \left\{\lambda' S_{sp}(\lambda') \Big/ \int_0^\infty \lambda S_{sp}(\lambda) d\lambda\right\}^{n+1} d\lambda'} = \frac{\left\{\lambda S_{sp}(\lambda)\right\}^{n+1}}{\int_0^\infty \left\{\lambda' S_{sp}(\lambda')\right\}^{n+1} d\lambda'}. \quad \text{(S7)}$$

The normalized contribution of the outgoing $n+1$-fold photon packets at the wavelength of λ to the spectral density of the fluorescence output can be expressed as

$$\tilde{S}_{n+1}(\lambda) = \frac{S_{n+1}(\lambda)}{\int_0^\infty S_{n+1}(\lambda) d\lambda} = \left(\frac{1}{\lambda}\right) \cdot p_{n+1}(\lambda) = \left(\frac{1}{\lambda}\right) \cdot \frac{\left\{\lambda S_{sp}(\lambda)\right\}^{n+1}}{\int_0^\infty \left\{\lambda' S_{sp}(\lambda')\right\}^{n+1} d\lambda'}. \quad \text{(S8)}$$

In the framework of the considered approach, the spectrum of the total fluorescence output can be obtained using an incoherent summation of partial contributions associated with the photon packets at the given wavelengths, which undergo various numbers of successive interactions with excited fluorophore molecules. Accordingly, the normalized spectral density of the fluorescent response can be written as

$$\tilde{S}(\lambda) = \sum_{n=0}^{\infty} \tilde{S}_{n+1}(\lambda) p_s(n); \sum_{n=0}^{\infty} p_s(n) = 1. \quad \text{(S9)}$$

Here, $p_s(n)$ is the discrete probability for finding a $n+1$-fold photon packet in the ensemble of detected packets. Taking into account a continuous character of the pathlength distributions $\rho(s)$ of fluorescence photons randomly propagating in the pumped medium, we modify Eq. S9 to the following form

$$\tilde{S}(\lambda) = \int_0^\infty \tilde{S}_{\frac{s}{l_{st}}+1}(\lambda)\,\rho(s)\,ds; \int_0^\infty \rho(s)\,ds = 1, \qquad \text{(S10)}$$

where the subscript $s/l_{st}+1$ takes non-integer values.

References

S.1. Ishimaru, A. *Wave Propagation and Scattering in Random Media*; Academic Press, New York, USA, 1978; V. 1, pp. 41-167.

S.2. Garcia, N.; Genack, A.Z.; Lisyansky, A.A. Measurement of the transport mean free path of diffusing photons. *Phys. Rev. B* **1992**, *46*, 14475-14479.

S.3. Zimnyakov, D.A.; Volchkov, S.S.; Kochkurov, L.A.; Kochubey, V.I.; Melnikov, A.G.; Melnikov, G.V. Speckle pattering of a pumping laser light as a limiting factor for stimulated fluorescence emission in dense random media. Opt. Express 2021, 29, 2309–2331.

S.4. Rivas, J.G.; Sprik, R.; Soukoulis, C.M.; Busch, K.; Lagendijk, A. Optical transmission through strong scattering and highly polydisperse media. *Europhys. Lett.* **1999**, *48*, 22–28.

S.5. Rivas, J.G.; Sprik, R.; Lagendijk, A.; Noordam, L.D.; Rella, C.W. Static and dynamic transport of light close to the Anderson localization transition. *Phys. Rev. E* **2001**, *63*, 046613.

S.6. Zhu, J.X.; Pine, D.J.; Weitz, D.A. Internal reflection of diffusive light in random media. *Phys. Rev. A* **1991**, *44*, 3948–3959.

S.7. Busch, K.; Soukoulis, C.M.; Economou, E.N. Transport and scattering mean free paths of classical waves. *Phys. Rev. B* **1994**, *50*, 93–98.

S.8. Busch, K.; Soukoulis, C.M. Transport properties of random media: an energy-density CPA approach. *Phys. Rev. B* **1996**, *54*, 893–899.

S.9. Zimnyakov, D.A.; Pravdin, A.B.; Kuznetsova, L.V.; Kochubey, V.I., Tuchin, V.V.; Wang, R.K.; Ushakova, O.V. Random media characterization using the analysis of diffusing light data on the basis of an effective medium model. *JOSA A* **2007**, *24*, 711-723.

S.10. Bohren, C.F.; Huffman, D.R. *Absorption and Scattering of Light by Small Particles*; Wiley, New York, USA, 1983; pp. 101-122.

S.11. Saulnier, P.M.; Zinkin, M.P.; Watson, G.H. Scatterer correlation effect on photon transport in dense random media. *Phys. Rev. B* **1990**, *42*, 2621–2623.

S.12. Oregon Medical Laser Center. Mie Scattering Calculator. Available online: https://omlc.org/calc/mie_calc.html (accessed on 10 June 2022).
